# A synopsis of eukaryotic N^α^-terminal acetyltransferases: nomenclature, subunits and substrates

**DOI:** 10.1186/1753-6561-3-S6-S2

**Published:** 2009-08-04

**Authors:** Bogdan Polevoda, Thomas Arnesen, Fred Sherman

**Affiliations:** 1 Department of Biochemistry and Biophysics, University of Rochester Medical Center, Rochester, NY 14642, USA; 2 Department of Molecular Biology, University of Bergen, N-5020 Bergen, Norway; 3 Department of Surgical Sciences, University of Bergen, N-5020 Bergen, Norway; 4 Department of Surgery, Haukeland University Hospital, N-5021 Bergen, Norway

## Abstract

We have introduced a consistent nomenclature for the various subunits of the NatA-NatE N-terminal acetyltransferases from yeast, humans and other eukaryotes.

## Introduction

**Figure 1:**
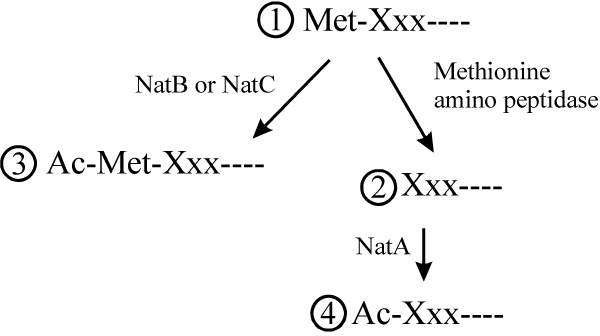
**A summary of the major pathways of *N*-terminal processing in eukaryotes, showing the four different termini**. 1: Uncleaved and unacetylated Met-Xxx- N-termini; 2: Cleaved and unacetylated Xxx-N-termini; 3: Uncleaved and NatB/NatC acetylated Ac-Met-Xxx- N-termini; 4: Cleaved and NatA acetylated Ac-Xxx-N-termini. See Table 1 and Figure 2 for more detail.

**Figure 2:**
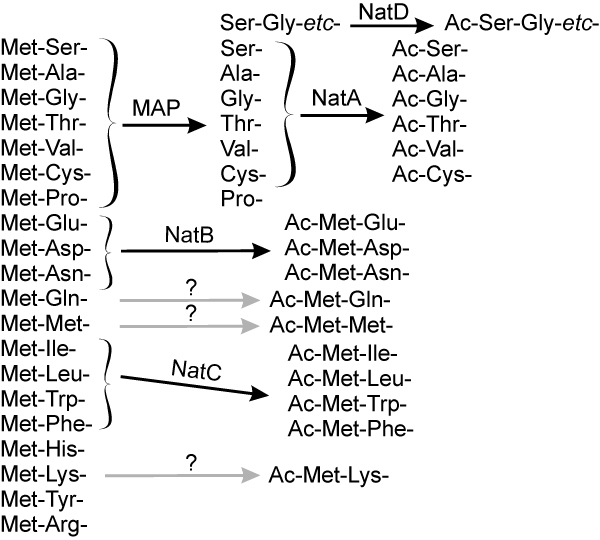
**The major pathways of *N*-terminal processing in eukaryotes**. Two methionine aminopeptidases (MAP), Map1p and Map2p, cleave *N*-terminal methionine residues that have small side chains (glycine, alanine, serine, cysteine, threonine, proline, and valine), although methionine is retained on some proteins having penultimate residues of valine. Subsequently, NatA, NatB, and NatC acetylate specific sequences as shown in the figure and in Table 1. Acetylation occurs at least partially on all proteins with Met-Glu-, Met-Asp- and Met-Asn- termini, but only on subclasses of proteins with the other termini. For example, acetylation occurs at least partially on 43% of proteins in yeast and on 96% of proteins in humans with Ala- termini. In addition, Ac-Cys-, Ac-Val-, Ac-Met-Met-, and Ac-Met-Lys- termini occurs on some proteins from humans but not from yeast; it is unknown which NATs are responsible for Ac-Cys-, Ac-Met-Met-, and Ac-Met-Lys- acetylations.

N-terminal acetylation has been extensively studied in yeast and humans and represents one of the most common protein modifications in eukaryotes, occurring on approximately 57% of yeast proteins and 84% human proteins [1], although it is rare in prokaryotes. Eukaryotic proteins initiate with methionine residues, which are cleaved from nascent chains if the penultimate residue has a radius of gyration of 1.29 Å or less [2]. *N*-terminal acetylation subsequently occurs on certain of the proteins, either containing or lacking the methionine residue, as depicted in Fig. 1. The salient features of *N*-terminal acetylation are summarized in Table 1 and Fig. 2. Detailed reviews on the N-terminal acetyltransferases have appeared [3-7], and the N-terminal acetylation status of 742 human and 616 yeast protein N-termini have been compiled [1]. The wide range and diversity of substrates is due in part to the large number of different *N*-terminal acetylating enzymes, NatA-NatE. The sequence requirements for *N*-terminal acetylation vary with the *N*-terminal acetyltransferase. Only two amino acid residues, Met-Asn-, Met-Asp-, or Met-Glu-, are required for at least partial *N*-terminal acetylation by NatB [1,8]. On the other hand, 30 to 50 specific amino acids are required for *N*-terminal acetylation by NatD [9]. Each of the three major *N*-terminal acetyltransferases, NatA, NatB and NatC, contain a catalytic subunit, and one or two auxiliary subunits (Table 1). The sequence and functions of the yeast and human orthologous subunits are obviously related. A yeast *ard1*-Δ *nat1*-Δ strain was phenotypically complemented by h*ARD1 *h*NAT1*, suggesting that yNatA and hNatA are similar. However, heterologous combinations, h*ARD1 *y*NAT1 *and y*ARD1 *h*NAT1*, were not functional in yeast, suggesting significant structural subunit differences between the species [1].

**Table 1:** Revised nomenclature for N-terminal acetyltransferases

Type	NatA	NatB	NatC	NatD	NatE
Original					
Catalytic subunit	Ard1p	Nat3p	Mak3p	Nat4p	Nat5p
Auxiliary subunit	Nat1p	Mdm20p	Mak10p		†
			Mak31p		

Revised					
Catalytic subunit	Naa10p	Naa20p	Naa30p	Naa40p	Naa50p
Auxiliary subunit	Naa15p	Naa25p	Naa35p		†
			Naa38p		

Number of yeast substrates	~2,000	~1,000	~250	2?	?

Substrates*	Ser-	Met-Glu-	Met-Ile-	Ser-Gly-*etc*-	?
	Ala-	Met-Asp-	Met-Leu-		
	Gly-	Met-Asn-	Met-Trp-		
	Thr-		Met-Phe-		
	Val-‡				
	Cys-¶				
	------------2 to 8 amino acids-----------			30–50 a. a	?

Naa50p is inferred to be an N-terminal acetyltransferase because of its sequence homology to known NATs.

* Acetylation occurs at least partially on all proteins with Met-Glu-, Met-Asp- and Met-Asn- termini, but only on subclasses of proteins with the other termini.

† Naa15p may be an auxiliary subunit of NatE, as well as an auxiliary subunit of NatA.

‡ Found in humans but not yeast (see Figure 2 legend).

¶One example found in yeast (see Figure 2 legend).

## Nomenclature

During a recent international meeting on N-terminal acetylation, it was pointed out that there is critical need to revise the gene symbols encoding the N-terminal acetyltransferases. The main reason for changing the nomenclature is so that each of the orthologous genes from different species would have the same name. Furthermore, orthologous genes were assigned not only by similarity of their sequences, but also by their action on the same set of proteins. Yeast NatA and human NatA were shown to acetylate the same set proteins by comparing a normal yeast strain with the mutant *naa10*-Δ *naa15*-Δh*NAA10 *h*NAA15 *[1].

The use of the different symbols *NAT*, *ARD*, *MDM*, and *MAK *is confusing, and does not provide useful information, especially when applied to human NATs. We believe it can be misleading to assign a gene symbol based on one phenotype of a mutant when a large number of proteins are affected, and when the mutant is pleiotropic.

Most importantly, different orthologous genes should have different names. The symbols *NAT1*, *NAT2 *and *NAT3 *denote human genes encoding arylamine N-acetyltransferases, which are distinct from N-terminal acetyltransferases [10]. On the other hand, NCBI has designated the human homologue of the yeast *NAT *genes as follows: y*NAT1 *designated as h*NARG1*; y*NAT3 *designated as h*NAT5*; and y*NAT5 *designated as h*NAT13*. Also, *ARD1 *is used to describe the ADP-ribosylation factor domain protein 1 [11].

Therefore, in this paper we have introduced a new nomenclature for protein *N*-terminal acetyltransferases in eukaryotes (Table 1). It is important to note that *NAA *(Nα acetyltransferases) is not used to designate any other gene in yeast or higher eukaryotes. We have assigned each of the subunits of the NatA-NatE complexes a Naa symbol, as presented in Table 1. We have also recommended a nomenclature for paralogs of human NatA complexes containing either Naa10p or Naa11p in combination with either Naa15p or Naa16p (Table 2). The revised symbols, along with synonyms from yeast and humans, are presented in Table 3. Clearly, this revised nomenclature will greatly diminish the confusion in describing orthologous subunits from different species.

**Table 2:** Paralogs

Subunit	Complex
Catalytic subunit Naa10p, Naa11p	
	NatA(10+15); NatA(10+16); NatA(11+15); NatA(11+16)
Auxiliary subunit Naa15p, Naa16p	

Almost all human NAT subunit genes encode alternative splicing isoforms whose functions are in question, and are not considered here.

**Table 3:** Synonyms

		Accession no.		
			
Primary name	Synonyms	Yeast	Human	References
Naa10p	Ard1p; TE2	P07347	P41227	[12-14]
Naa11p	Ard2p	-	Q9BSU3	[15]
Naa15p	Nat1p; NARG1; NATH; TBDN	P12945	Q9BXJ9	[16-20]
Naa16p	Nat2p; NARG1L	-	Q6N069	[20,21]
Naa20p	Nat3p; hNat5p	Q06504	P61599	[8,22,23]
Naa25p	Mdm20p; p120	Q12387	Q14CX7	[8,23]
Naa30p	Mak3p; hNat12p	Q03503	Q147X3	[24-26]
Naa35p	Mak10p; hEGAP	Q02197	Q5VZE5	[24,25,27]
Naa38p	Mak31p; hLsm8p	P23059	O95777	[24,25,27]
Naa40p	Nat4p; hNat11p	Q04751	Q86UY6	[28]
Naa50p	Nat5p; hNat13p; San	Q08689	Q9GZZ1	[29-31]

An example of standard symbols: Protein, Naa10p; Gene, *NAA10*; Deleted gene, *naa10-Δ*. hNaa10p, human; yNaa10p, yeast (*S. cerevisiae*); mNaa10p, mouse.

## Competing interests

The authors declare that they have no competing interests.

## Authors' contributions

All authors wrote the manuscript and approved the final version.
